# PD-1 inhibitor-based adverse events in solid tumors: A retrospective real-world study

**DOI:** 10.3389/fphar.2022.974376

**Published:** 2022-11-09

**Authors:** Guili Huang, Songqing Liu, Jie Dong, Xin Xi, Rui Kong, Wenjun Li, Qian Du

**Affiliations:** ^1^ Department of Pharmacy, The Third Affiliated Hospital of Chongqing Medical University, Chongqing, China; ^2^ Department of Oncology, The Third Affiliated Hospital of Chongqing Medical University, Chongqing, China

**Keywords:** immune checkpoint inhibitor, PD-1 inhibitors, irAEs, real-world study, solid tumors

## Abstract

**Background & Aims:** Immune checkpoint inhibitors (ICIs) have transformed the landscape of cancer treatment, and ICI-related toxicities (i.e., immune-related adverse events (irAEs) have been reported in many clinical studies. However, the toxicity data of real-world have not been fully assessed.

**Methods:** Patients with histologically confirmed solid tumors who had been treated with PD-1 inhibitors were included in the study. Patient data were collected from electronic medical records, including basic characteristics, data of irAEs, management and outcome. Incidences of irAEs were pooled and compared, and the risk of irAEs was also analyzed.

**Results:** A total of 362 solid tumor patients treated with sintilimab (*n* = 171), camrelizumab (*n* = 60), toripalimab (*n* = 72), and pembrolizumab (*n* = 59) were included. In total, any grade irAEs, grade 1–2 irAEs, and grade ≥3 irAEs accounted for 47.24%, 38.67% and 8.56% of cases, reapectively. Further, 29.24% of patients discontinued immunotherapy due to irAEs, with pneumonitis being the main reason for discontinuation. By comparing the toxicity profiles between different ICIs, we found that reactive capillary haemangiomas were camrelizumab-specific. Additionally, the frequency of irAEs was association with ICIs type, the pooled incidence (standardized rate) of irAEs related to sintilimab, camrelizumab, toripalimab and pembrolizumab were 55.56% (52.81%), 48.33% (55.55%), 33.33% (29.23%) and 38.98% (38.29%), respectively. Sintilimab and camrelizumab had higher incidences of any grade and grade 1–2 than toripalimab (55.56% vs. 33.33%, *p* = 0.002; 48.54% vs. 25.00%, *p* = 0.0001) and pembrolizumab (55.56% vs. 38.98%, *p* = 0.0028; 48.54% vs. 25.42%, *p* = 0.002), while the grade ≥3 irAEs of pembrolizumab (13.56%) were approximately 1.63- to 1.93-fold higher than other ICIs, and the standardized grade ≥3 of pembrolizumab was significantly higher than that of sintilimab (13.21% vs. 7.12%, *p* = 0.026), especially for grade ≥3 pneumonitis. Multivariate analysis found that cumulative cycles of ICI (OR = 1.081; 95% CI: 1.023–1.142; *p* = 0.006), and lung cancer (OR = 1.765; 95% CI: 1.105–2.820; *p* = 0.017) were independent risk factors for irAEs.

**Conclusion:** The frequency of irAEs is associated with ICI type. The pooled incidence of irAEs related to sintilimab and pneumonitis caused by pembrolizumab were higher. These data indicate the importance of having different monitoring priorities for different PD-1 inhibitors.

## Introduction

Over the past 5 years, immune therapy represented by immune checkpoint inhibitors (ICIs) targeting programmed death 1 (PD-1), programmed death ligand-1 (PD-L1) and cytotoxic T lymphocyte-associated antigen-4 (CTLA-4), have been important therapeutic drugs second only to chemotherapy, radiotherapy and targeted therapy, because ICIs have significantly improved clinical outcomes in multiple cancer types (Vaddepally et al., 2020). Different from conventional cytotoxic drugs, ICIs restore and promote antitumor immunity to kill cancer cells (Thompson et al., 2022). Currently, ICIs (e.g., nivolumab, pembrolizumab, atezolizumab, camrelizumab, toripalimab, tislelizumab and sintilimab) alone or combined with chemotherapy or anti-angiogenesis therapy have become the standard first- or second-line therapies for various malignancies. Moreover, domestic ICIs (camrelizumab, toripalimab, tislelizumab and sintilimab) are used more than abroad ICIs (pembrolizumab and nivolumab) because of drug costs.

With the widespread use of immunotherapy, the development of ICI-related toxicities (i.e., immune-related adverse events, irAEs) are concerning because many like myocarditis and pneumonitis are covert and fatal (Wang et al., 2018; Chen et al., 2021; Luo et al., 2021). Different from the adverse reactions mediated by traditional cytotoxic drugs, irAEs are associated with an immunologic mode of action, which can affect any organ system in the body. The most frequently organ include of skin, endocrine system, lungs, heart, gastrointestinal (GI) system, liver and other less common inflammatory events (Xu et al., 2018; Yang et al., 2019). Moreover, the characteristics of irAEs are not typical. The frequency of irAEs depends on the type of ICIs as well as the tumor type, the duration of exposure, the dose administered, and the specific characteristics of individual patients (e.g., such as intrinsic risk factors (Ma et al., 2018; Pillai et al., 2018; Tang et al., 2021). The results of a recent meta-analysis showed that GI toxicity and hypophysitis are more frequent with CTLA-4 inhibitors, whereas pneumonitis, hypothyroidism, arthralgia, and thyroid disorders are more prevalent with PD-1 inhibitors (Boutros et al., 2016; Choi and Lee, 2020). Patients receiving PD-1/PD-L1 inhibitors have a lower incidence of any-grade irAEs than those receiving CTLA-4 inhibitors, while patients receiving a combination of PD-1/PD-L1 inhibitors and CTLA-4 inhibitors have the highest incidence of irAEs. The incidence of grade ≥3 irAEs is 6%, 24% and 55% for patients receiving PD-1/PD-L1 inhibitors, CTLA-4 inhibitors, combination of the two immunotherapy, respectively (Haanen et al., 2017; Martins et al., 2019). Some systematic reviews have also shown that patients with non-small cell lung cancer (NSCLC) experience a higher frequency of pneumonitis and a lower frequency of GI and skin irAEs compared to melanoma (Khoja et al., 2017). Moreover, irAEs have very wide dispersion of onset times, with data from treatment process to 1 year after discontinuation (Zhai et al., 2019). These nonspecific features make it difficult to identify adverse reactions, and limit clinical applications.

Although many studies have reported on ICI-related toxicities, the toxicity data are mainly from clinical research which cannot fully substitute for real-world ICI-related toxicities due to certain limitations. Therefore, there is an urgent clinical need to obtain real-world safety data. Herein, we evaluate the real-world characteristics and potential risk of irAEs, with the aim of improving early recognition and management of irAEs and to also provide a reference for the use of ICIs.

## Methods

We performed a retrospective observational study to investigate the characteristics of adverse drug reactions (ADRs) related to ICI, in a tertiary hospital (Chongqing, China) between January 2018 and July 2021. Informed consent was permitted obtained from all study participants. The inclusion criteria were as follows: (Vaddepally et al., 2020): patients were diagnosed as solid malignant tumors with pathologic diagnosis; (Thompson et al., 2022); patient with ≥2 cycle of ICI therapy (pembrolizumab, sintilimab, camrelizumab, tirelizumab, or toripalimab). The dosage of ICI was given referring to prescribing information and guidelines, such as pembrolizumab 2 mg/kg or 200 mg iv q3w, sintilimab 200 mg iv q3w, camrelizumab 200 mg iv q2w or q3w, tirelizumab 200 mg iv q3w, or toripalimab 3mg/kg iv q2w or 240 mg iv q3w. Patients with incomplete medical records were excluded. In our study, irAEs were defined according to the guidelines on the management of immunotherapy-related toxicities (Thompson et al., 2022) and finally diagnosed by medical specialists. The grade of irAEs was determined using the Common Terminology Criteria for Adverse Events version 5.0.

### Data collection

Available medical records in each case were reviewed for the patient’s medical history, including age, sex, history of disease, the type of tumor, duration of ICI therapy, and details of other chemotherapies and radiotherapy. The data of adverse reaction included onset time, symptoms, auxiliary examination, and the details of treatment approaches for ICI-related adverse reactions. Patient outcomes were also recorded.

### Statistical analysis

The major endpoint of our study was to observe the characteristics of irAEs in real study, including irAE profiles, classification of adverse reactions, occurrence time, management and outcomes. We also sought to compare irAE profiles between different ICIs. Other assessed outcomes included an exploration of exploring risk factors associated with irAEs. Data are presented with descriptive statistics for each demographic. Association studies were conducted using Fisher’s exact test for categorical variables and the Mann-Whitney *U* test for continuous variables. Multivariate logistic regression analysis was conducted to identify potential risk factors for irAEs. The risk associations with a *p*-value < 0.05 were considered statistically significant, and all analyses were performed using SPSS. In addition, the incidence of different ICIs was standardized using the standardized equation ( $P^{\prime }=\left(⅀NiPi\right)/N$ **)** to reduce the bias, when there was significant difference in the composition ratio between different ICIs.

## Results

### Patient characteristics

From January 2018 to July 2021, 441 patients were treated with ICIs (Pembrolizumab, Sintilimab, Camrelizumab, Tirelizumab, or Toripalimab). A total of 79 patients were excluded because they received one treatment cycle, therefore, the study cohort consisted of 362 patients. The 362 patients received 1,256 treatment cycles. Of the 362 patients with ICI therapy, 275 (75.97%) were male, giving a male: female ratio of 3.16:1. The average age was 62.4 ± 11.4 years (range 11–86 years). Lung cancer was the most type of cancer (201 patients, 55.52%), followed by head and neck cancer (61 patients, 16.85%), and liver cancer (34 patients, 9.39%). The other details of the basic information are shown in Table 1, and the baseline information of people with different ICIs is also shown in Supplementary Table S1 of Supplementary Material. There were no significant differences among different ICI groups in terms of age, sex, smoking history, chronic obstructive pulmonary disease, medication information, targeted therapy history and radiation history; only the composition of tumor type was statistically significant (*p* = 0.001).

**TABLE 1:** Clinical characteristics of patients with ICIs.

Characteristics	Patients (*n* = 362)	Characteristics	Patients (*n* = 362)
Sex		Treatment	
Male	275 (75.97%)	Monotherapy	60 (16.57%)
Female	87 (24.03%)	Chemotherapy	201 (55.52%)
Age (year)		anti-vascular drug	85 (23.48%)
<65	193 (53.31%)	anti-vascular and chemotherapy	16 (4.42%)
> =65	169 (46.69%)	Line of therapy	
Disease history		1 line	216 (59.67%)
0	156 (43.09%)	> =2 line	146 (40.33%)
1–2	184 (50.83%)	Past medical history	
> =3	22 (6.08%)	Autoimmune condition	3 (0.83%)
Type of cancer		Targeted therapy history	12 (3.31%)
Lung	201 (55.52%)	Radiation history	33 (9.12%)
head and neck	61 (16.85%)	Cumulative cycles	
Liver	34 (9.39%)	≤4	213 (58.84%)
Other	66 (18.23%)	>4	149 (41.16%)

### Incidence and spectrum of irAEs

**FIGURE 1:**
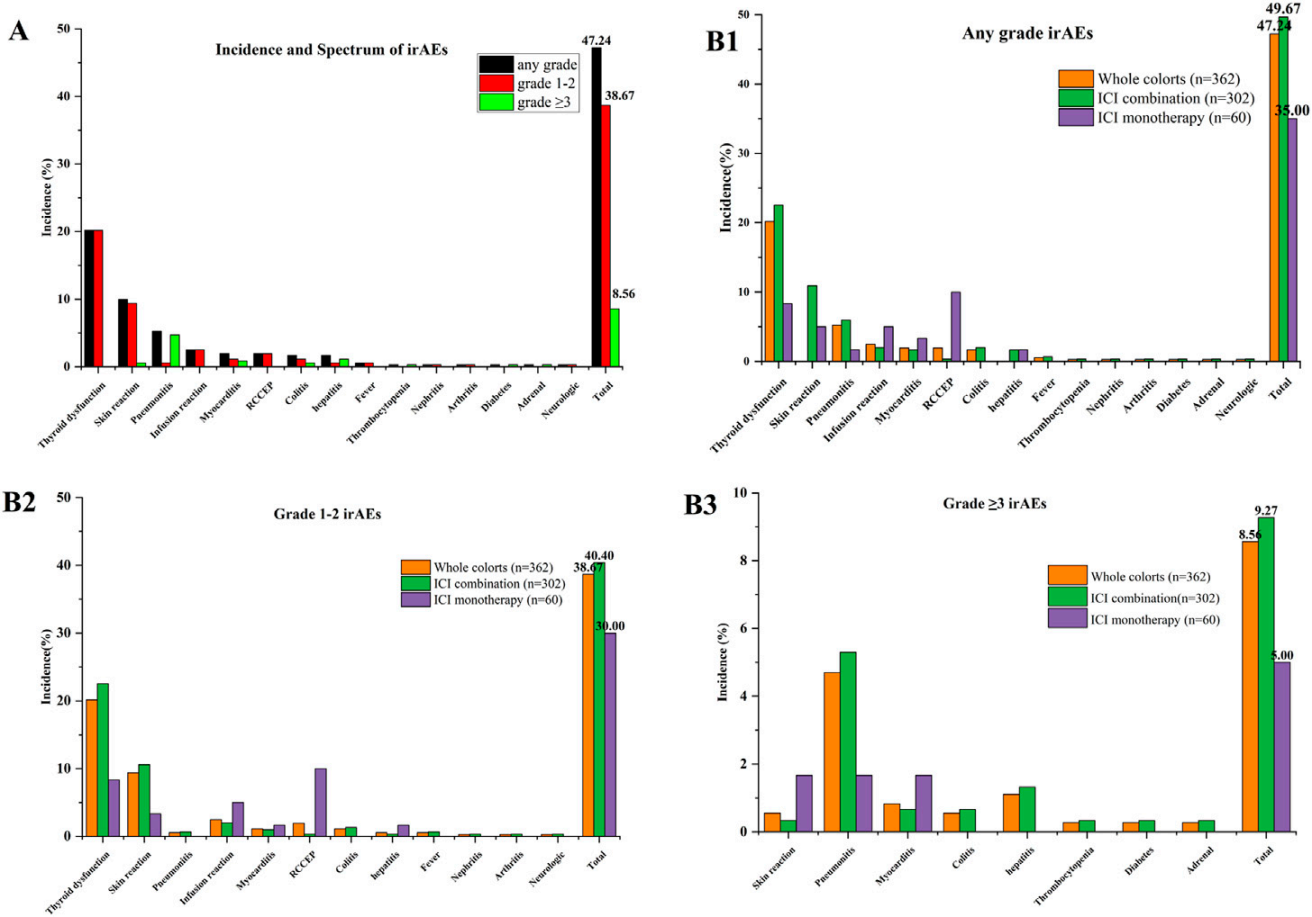
Incidence and spectrum of irAEs between immune checkpoint inhibitor monotherapy and combination: **(A)** Incidence and spectrum of irAEs; **(B1–3)** irAEs between immune checkpoint inhibitor monotherapy and combination. ICI combination: combination with chemotherapy, anti-vascular drug, anti-vascular and chemotherapy.

**FIGURE 2:**
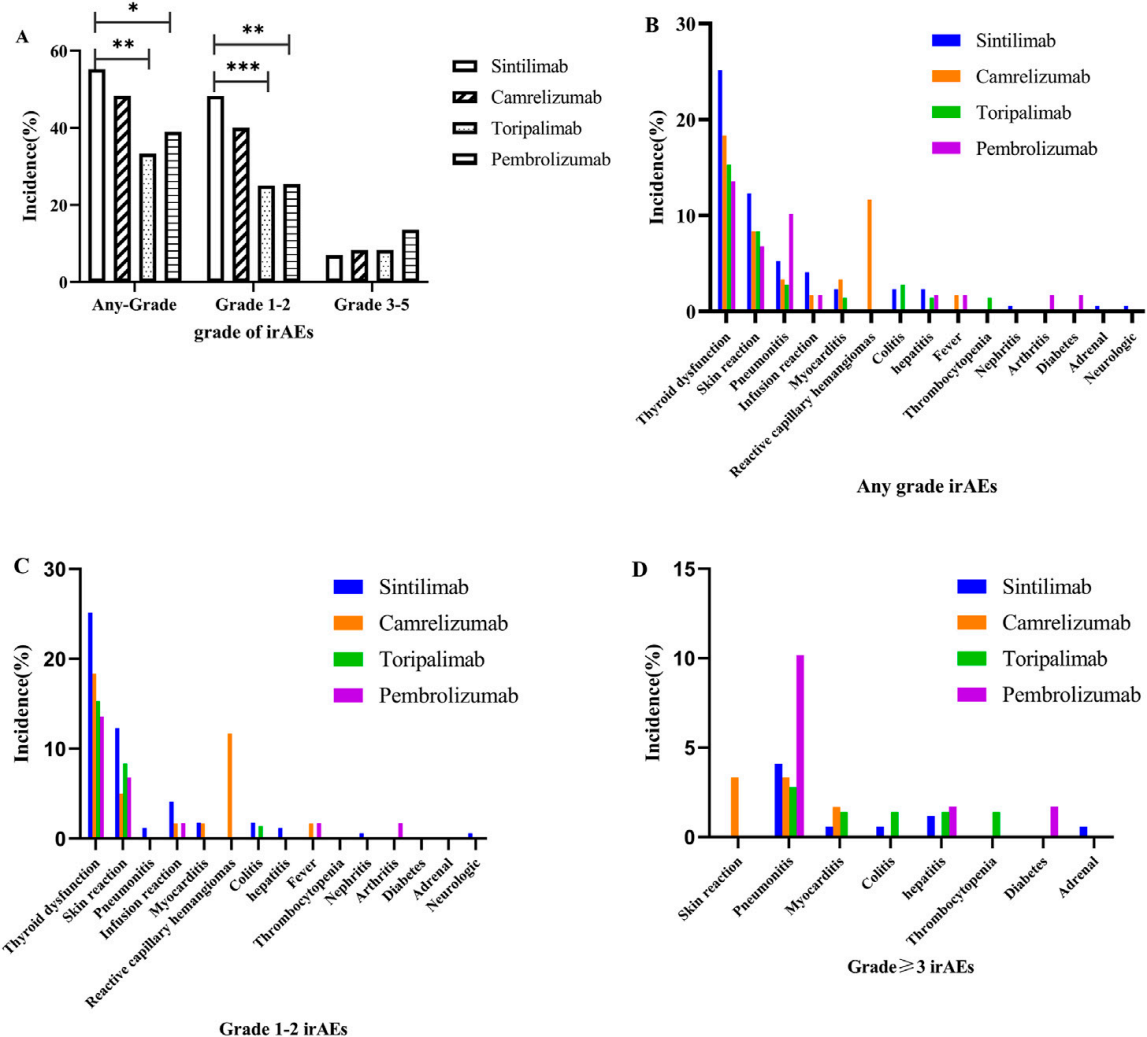
Immune-related adverse events between different immune checkpoint inhibitors: **(A)** any irAEs; **(B)** any grade; **(C)** grade 1–2; **(D)** grade ≥3.

**FIGURE 3:**
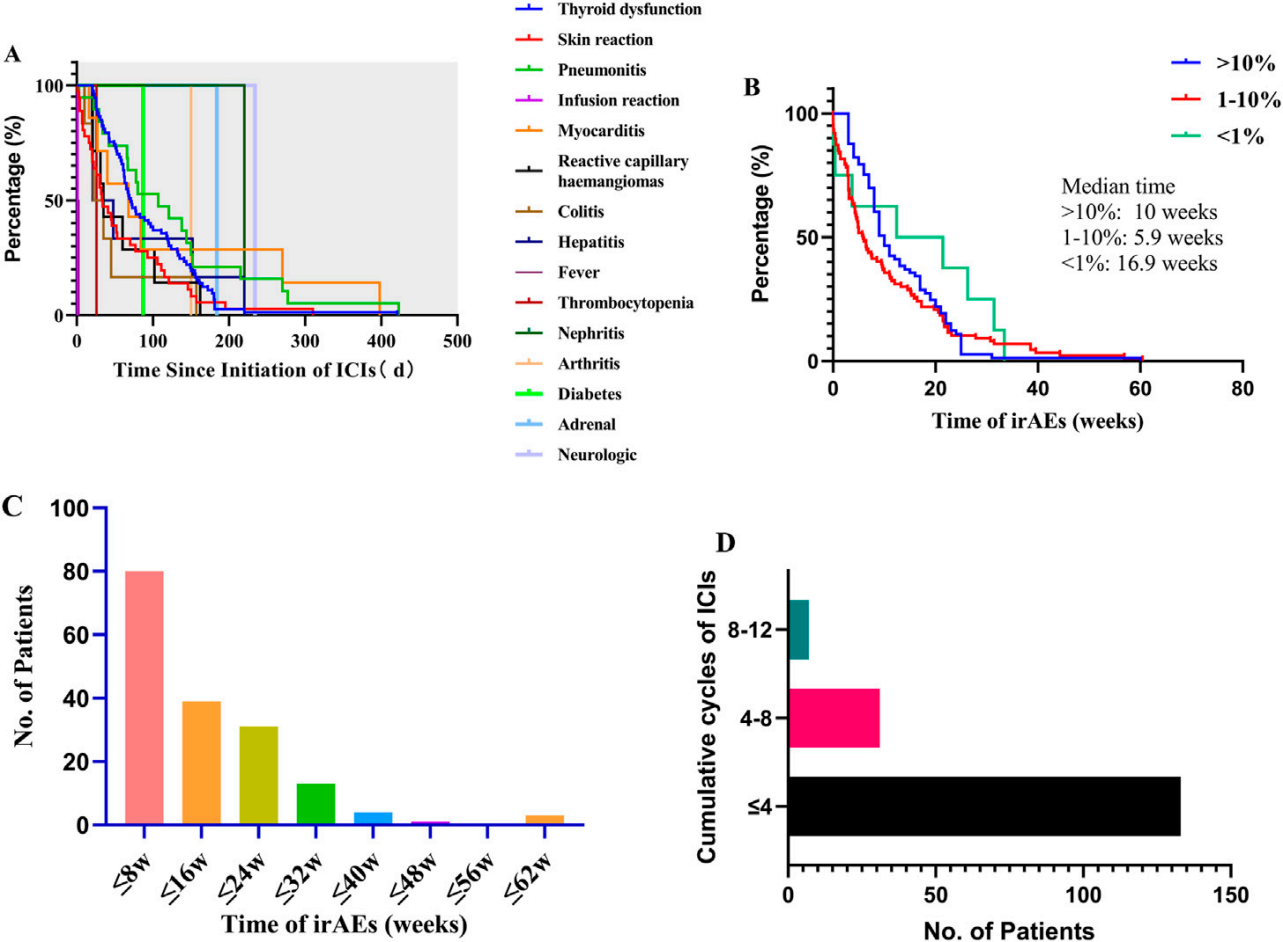
Onset time between different irAEs and frequency of all irAEs between onset time and treatment cycle: **(A)** onset time of any irAEs; **(B)** onset time between different frequency of irAEs; **(C)** frequency of all irAEs by onset time; **(D)** frequency of all irAEs by treatment cycle.

Of the 362 patients, 118 patients (32.6%) developed a total of 171 irAEs, among which 80 patients (67.80%) had one or two types of irAEs and nine patients (7.63%) suffered more than three kinds of irAEs. The common reported events (≥1%) by organ system were thyroid dysfunction (73, 20.17%), skin reaction (36, 9.94%), pneumonitis (19, 5.25%), infusion reaction (9, 2.49%), myocarditis (7, 1.93%), reactive capillary hemangiomas (7, 1.93%), colitis (6, 1.66%), and hepatitis (6, 1.66%). Moreover, rare irAEs (<1%) included fever (*n* = 2), thrombocytopenia (*n* = 1), nephritis (*n* = 1), arthritis (*n* = 1), diabetes (*n* = 1), adrenal (*n* = 1) and neurologic (*n* = 1), as listed in Supplementary Table S2 (Supplementary Material). Overall, the incidence of any grade irAEs, grade 1–2 irAEs, and grade ≥3 irAEs were 47.24%, 38.67%, and 8.56%, respectively. Further, irAEs were mostly mild, and there were significant differences between grade 1–2 and grade ≥3 (38.67% vs. 8.56%, *p* < 0.01) (Figure 1A, Supplementary Material, Supplementary Table S3). For grade ≥3 irAEs, the incidence of pneumonitis was the highest, reaching 4.7%, followed by hepatitis (1.1%), myocarditis (0.83%), skin reaction (0.55%), colitis (0.55%), thrombocytopenia (0.28%), diabetes (0.28%), and adrenal (0.28%). The toxicity data of different regimen were also revealed in Figure 1B, Figure 1, Figure 2, Figure 3 (Supplementary Material, Supplementary Table S3). These data showed that the ICI combination group had significantly higher rates of any grade irAEs (49.67% vs. 35%, *p* = 0.038) compared to the monotherapy group, while there were no significant differences between groups with grade 1–2 and grade ≥3 irAEs. Notably, the frequency of reactive capillary haemangiomas (RCCEP) were higher in the ICI monotherapy group than those in the ICI combination group (10% vs. 0.33%, *p* < 0.001).

We further analyzed and compared the incidence to ICI-related adverse reactions associated with the ICI types. Figure 2 and Supplementary Table S4A–C of the Supplementary Material show that there were no significant differences among the spectrum of the four ICIs (sintilimab, camrelizumab, toripalimab, and pembrolizumab). Interestingly only camrelizumab caused reactive capillary haemangiomas, indicating that this AE should be camrelizumab-specifc. Moreover, the four ICIs had different incidence of any grade, and the pooled incidence of sintilimab, camrelizumab, toripalimab and pembrolizumab was 55.56%, 48.33%, 33.33% and 38.98%, respectively. Sintilimab had signifcantly higher incidences of any grade and grade 1–2 irAEs than toripalimab (55.56% vs. 33.33%, *p* = 0.002; 48.54% vs. 25%, *p* = 0.0001), and pembrolizumab (55.56% vs. 38.98%, *p* = 0.0028; 48.54% vs. 25.42%, *p* = 0.002). Considering the significant differences among groups in the composition of cancer type as shown in Supplementary Table S1(Supplementary Material), the incidence of irAEs with different ICI. The standardized incidence of all-grade irAEs with sintilimab, camrelizumab, toripalimab, and pembrolizumab was 52.81%, 55.55%, 29.23% and 38.29%, respectively, and grade 1–2 irAEs was 45.69%, 44.99%, 21.26%, and 25.08%, respectively. Sintilimab and camrelizumab had higher all-grade (52.81%/55.55% vs. 29.23%/38.29%, *p* < 0.001) and grade 1–2 irAEs (45.69%/44.99% vs. 21.26%/25.08%, *p* < 0.001) than toripalimab and pembrolizumab. For grade ≥3 irAEs, there were significant differences between sintilimab and pembrolizumab (7.12% vs. 13.21%, *p* = 0.026). Notably, the pooled incidences of grade ≥3 irAEs to pembrolizumab (13.56%) were approximately 1.93-, 1.63- and 1.63-fold higher than sintilimab (7.02%), camrelizumab (8.33%) and toripalimab (8.33%), which suggests pembrolizumab may have a bigger impact on severe irAEs. Additionally, pembrolizumab had a higher incidence of grade ≥3 pneumonitis (10.17% for pembrolizumab, 4.09% for sintilimab, 2.33% for camrelizumab and 2.78% for toripalimab), and the camrelizumab-induced grade ≥3 infusion reactions (3.33%) should not be ignored. The observed and standardized incidence of irAEs are shown in Table 2 and Supplementary Table S4A–C of the Supplementary Material.

**TABLE 2:** irAEs of different ICI.

	Observed rate			Standardized rate		
	Any grade (%)	Grade 1–2 (%)	Grade ≥3 (%)	Any grade (%)	Grade 1–2 (%)	Grade ≥3 (%)
Sintilimab	55.56	48.54	7.02	52.81	45.69	7.12
Camrelizumab	48.33	40.00	8.33	55.55	44.99	10.55
Toripalimab	33.33	25.00	8.33	29.23	21.26	7.97
Pembrolizumab	38.98	25.42	13.56	38.29	25.08	13.21

### Onset time and ICIs using cycle of irAEs

Overall, the time to the start of any irAEs was variable (median: 62 d, range: 0–423 d), and there were significant differences between the onset time of different irAE (*p* < 0.001), such as infusion reaction (transfusion process), fever (median: 2 d, range: 1–3 d), thrombocytopenia (26 d), colitis (median: 28.5 d, range: 10–157 d), skin reaction (median: 33.5 d, range: 1–310 d), hepatitis (median: 34.5 d, range: 20–220 d), reactive capillary hemangiomas (median: 35 d, range: 21–162 d), myocarditis (median: 68 d, range: 16–398 d), diabetes (87 d), pneumonitis (median:107d, range: 3–423 d), arthritis (150 d), thyroid dysfunction (median:171 d, range: 21–421 d), nephritis (184 d), adrenal (220 d), and neurologic (234 d) as shown in Figure 3A. Interestingly, the onset time of irAEs was related to the incidence of irAEs and there were significant differences (*p* < 0.05) in the most common irAEs (>10%, median:10 weeks), common irAEs (1–10%, median:5.9 w), and rare irAEs (<1%, median:16.9 w) (Figure 3B). This suggested that we should play attention to rare irAEs for patients with using undergoing long-term ICI treatment. Moreover, we analyzed the incidence to ICI-related adverse reactions associated with the ICI using cycle. A total of 171 irAEs occurred in 80 patients, the majority of which were early in the treatment course (Figure 3C). In addition, 77.78% of these irAEs occurred in the first four cycles, 18.13% within four to eight cycles, and approximately 4.09% within 8–12 cycles (Figure 3D with increased medication time, the incidence of thyroid dysfunction, skin reaction, infusion reaction, and pneumonitis decreased, while the frequency of rare irAEs (e.g., nephritis, adrenal, and neurologic) increased.

### Management and outcomes

**FIGURE 4:**
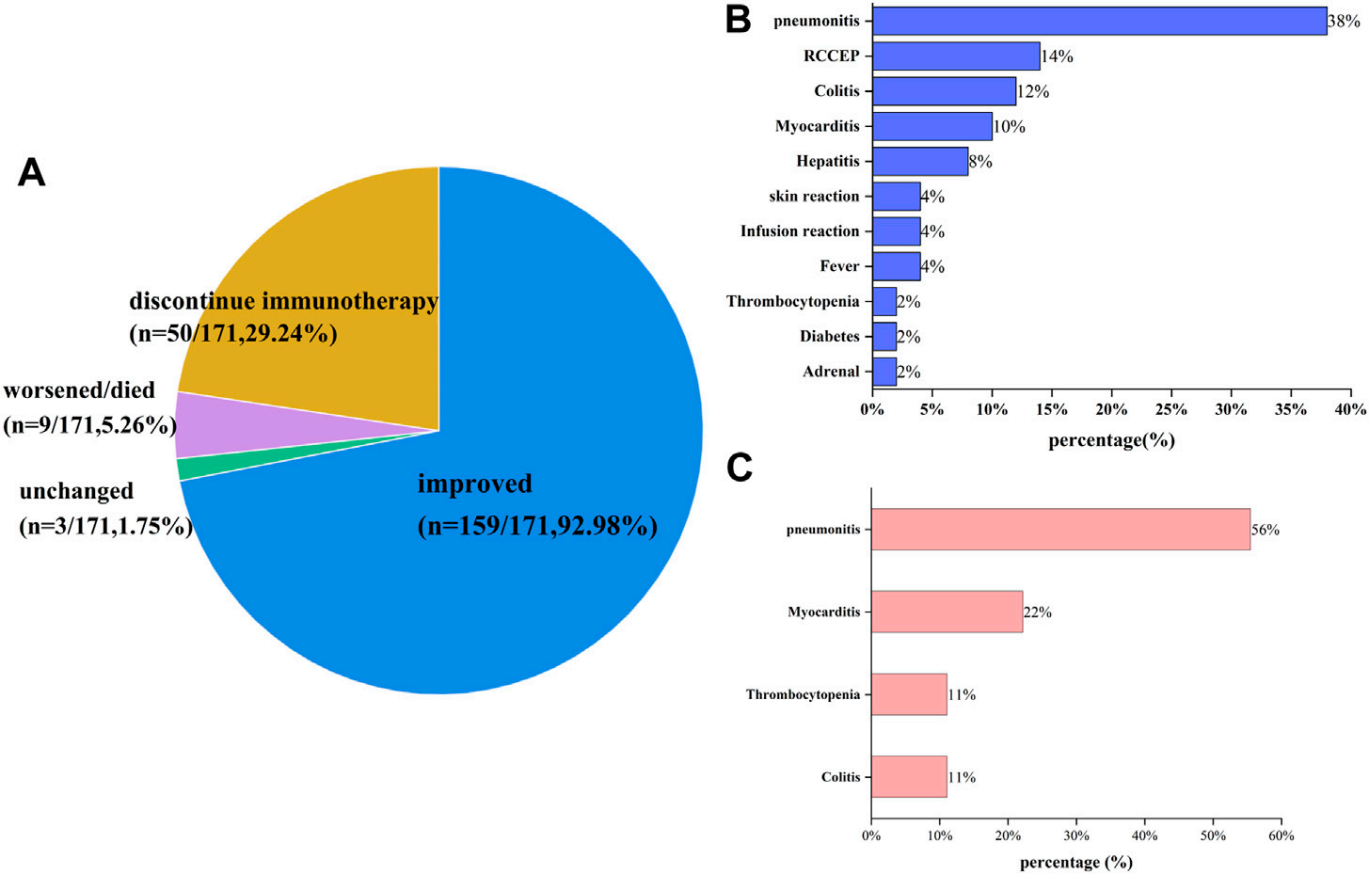
Outcomes of patients with irAEs: **(A)** the outcome distribution of patients with irAEs; **(B)** the reason for ending immunotherapy; **(C)** the cause of exacerbation or death for patients with irAEs. Overall, 92.98% of patients improved, and 1.75% of patients who experienced grade ≥3 RCCEP had unchanged. 5.26% of patients experienced worsened or died, with pneumonitis (*n* = 5/9, 55.56%), myocarditis (*n* = 2/9, 22.22%), colitis (*n* = 1/9, 11.11%) and thrombocytopenia (*n* = 1/9, 11.11%). 50 patients (29.24%) experienced discontinue immunotherapy for irAEs, with the common being pneumonitis (*n* = 19/50, 38%), reactive capillary hemangiomas (*n* = 7/50, 14%), colitis (*n* = 6/12, 12%), myocarditis (*n* = 5/50, 10%), and hepatitis (*n* = 4/50, 8%), and a few occurring in skin reaction (*n* = 2), infusion reaction (*n* = 2), fever (*n* = 2), thrombocytopenia (*n* = 1), diabetes (*n* = 1), and adrenal (*n* = 1).

The detailed clinical manifestations, management and outcomes of all irAEs are shown in Supplementary Table S5 of the Supplementary Material. These data demonstrated that most irAEs have no obvious clinical manifestations and patient outcomes were associated with the type and grade of irAEs. Overall, the mild or moderate irAEs of most patients were not associated with any apparent symptoms, and only showed abnormal laboratory results. Of the 171 irAEs, 159 had a good prognosis except for patients with thyroid dysfunction who required lifelong thyroid hormone replacement therapy. In addition, 1.75% (3 of 171) of patients were unchanged, and the health of 5.26% (9 of 171) patients worsened and some even died as shown in Figure 4A. We also found that 29.24% (50 of 171) discontinued immunotherapy because of their irAEs. In order of frequency, the irAEs of the nine patients whose health worsened and/or even died was pneumonitis (*n* = 5/9, 56%), myocarditis (*n* = 2/9, 22%), thrombocytopenia (*n* = 1/9, 11%) and colitis (*n* = 1/9, 11%) as shown in Figure 4B. Notably, 4 of 17 patients with grade ≥3 pneumonitis occurred rebound in the process of tapering off systemic steroids, while one patient died from pneumonitis; two of three patients with grade ≥3 myocarditis occurred rebound even died, and the patient with thrombocytopenia was neglected and died from delated treatment (Figure 4C). Moreover, ICI-related AEs leading to discontinuation were reported in 50 patients (29.24%), with the most common cause being pneumonitis (*n* = 19/50, 38%), reactive capillary hemangiomas (*n* = 7/50, 14%), colitis (*n* = 6/50, 12%), myocarditis (*n* = 5/10, 10%) and hepatitis (*n* = 4/10, 8%). Additionally, a few cases of discontinuation occurred following: skin reaction, infusion reaction, fever, thrombocytopenia, diabetes and adrenal. There were no instances of thyroid dysfunction.

### Risk factors for irAEs

In our study, we classified patients into two groups based on the prevalence of irAEs: those with irAEs (irAE group) and those without (non‐irAE group). Subsequently, we analyzed the susceptibility factors for irAEs using univariate and multivariate analyses. As shown in Table 3 and Table 4, univariate analysis revealed that there was a significant correlation between the incidences of irAEs and EGFR-TKI therapy history (*p* = 0.025), ICI cycles (*p* = 0.009), cancer types (*p* = 0.009) and Eos (*p* < 0.037). There were no significant differences between the incidence of irAEs and sex, age, treatment, line of therapy, or disease history. Using multivariate analysis, we found that cumulative cycles of ICI (OR = 1.081; 95% CI: 1.023–1.142; *p* = 0.006), and lung cancer (OR = 1.765; 95% CI: 1.105–2.820; *p* = 0.017) were independent risk factors for irAEs. Interestingly, the incidence of irAEs in patients, who had autoimmune diseases and had received radiotherapy in 90 d, did not markedly increase (*p* > 0.05) compared to the frequency of other patients.

**TABLE 3:** Risk factors for irAEs with univariate analysis.

	irAEs group (*n* = 118)	Non-irAEs group (*n* = 244)	*χ2*	*p*
Sex			3.037	0.081
Male	83 (70.34%)	192 (78.69%)		
Female	35 (29.66%)	52 (21.31%)		
Age (year)			2.538	0.111
<65	70 (59.32%)	123 (50.41%)		
> =65	48 (40.68%)	121 (49.59%)		
Smoking			0.600	0.439
Yes	69 (58.47%)	153 (62.70%)		
No	49 (41.53%)	91 (37.30%)		
chronic pulmonary disease			0.671	0.413
Yes	15 (12.71%)	39 (15.98%)		
On	103 (87.29%)	205 (84.02%)		
Treatment			0.595	0.441
ICIs monotherapy	17 (14.41%)	43 (17.62%)		
ICIs combinations	101 (85.59%)	201 (82.38%)		
Line of therapy			0.132	0.716
1 line	72 (61.02%)	144 (59.02%)		
≥2 line	46 (38.98%)	100 (40.98%)		
Past medical history				
Targeted therapy history	8 (6.78%)	4 (1.64%)	6.557	0.025
Radiation history	10 (8.47%)	23 (9.43%)	0.087	0.768
Autoimmune condition	1 (0.85%)	2 (0.82%)	0.001	0.978
Cumulative cycles			6.783	0.009
≤4	58 (49.15%)	155 (63.52%)		
>4	60 (50.85%)	89 (36.48%)		
Disease history			2.305	0.316
0	47 (39.83%)	109 (44.67%)		
1–2	66 (55.93%)	118 (48.36%)		
> =3	5 (4.24%)	17 (6.97%)		
Type of cancer			6.711	0.009
Lung	77 (65.25%)	124 (50.82%)		
Other	41 (34.75)	120 (49.18)		
Biomarkers				
Lymphocytes	1.22	1.26	—	0.915
Eosinophils	0.22	0.17	—	0.037

**TABLE 4:** Multivariable Logistic regression analysis for irAEs.

Risk	Β	S.E	Wald	P	OR	95% *CL*
Cumulative cycles	0.078	0.028	7.655	0.006	1.081	1.023–1.142
Lung cancer	0.568	0.239	5.654	0.017	1.765	1.105–2.820

## Discussion

With the expansion of the clinical indications for ICIs, their safety is a key factor for their broad clinical application. Due to the specific pharmacodynamics and pharmacokinetics of ICI immunotherapy, ICI-related toxicities are distinct from those observed with cytotoxic chemotherapy or targeted anti-cancer therapy. ICIs are capable of causing immune-related toxicity in almost all tissues. However, the prevalence and profile of ICI-related toxicity are not fully understanded and still study. The reported overall incidence of irAEs ranges widely from approximately 15–90% and is associated with ICI agent, tumor type, treatment lines of ICI initiation and derived neutrophil-to-lymphocyte ratio. These irAEs are most commonly found to affect the skin, GI tract, hepatitis, thyroid dysfunction, pneumonitis, and, rarely, myocarditis and the nervous system (Haanen et al., 2017). However, the data of ICI-related adverse reactions is mainly based on clinical trials and meta-analyses, which cannot substitute real-world treatment settings.

To our knowledge, there have been few retrospective observational studies to evaluate the real-world incidence, profile, management and risk factors of irAEs. Our analysis has revealed several important findings. We did not observe any novel irAEs but we did find that RCCEP was only reported in camrelizumab-treated patients, and that the incidence of RCCEP was lower in ICIs combination group (chemotherapy or anti-vascular drug). Further, we demonstrate that RCCEP is camrelizumab-specific as reported (Li et al., 2020). RCCEP is a highly specific off-target binding event, and the exact mechanisms of camrelizumab-induced RCCEP remain unknown. One possible explanation may be an imbalance in receptor/receptor-ligand interactions along with upregulation of vascular proliferative proteins (VEGF) (Teng et al., 2019). Camrelizumab, a potent agonist of human VEGFR-2, can drive hemangioma development *via* vascular endothelial cell activation and lead to high frequency of capillary hemangioma (Finlay et al., 2019). Anti-vascular drugs can block the VEGF-VEGFR signaling pathway, inhibit vascular cells, and lead to a decrease in the density of capillaries, which may explain why the incidence of RCCEP decreases for patients with camrelizumab and anti-vascular drugs (Li et al., 2019; Wang et al., 2021).

By indirectly comparing the toxicity data, we found that the prevalence of irAEs in different organs differed from those previously reported. Colitis and hepatitis were infrequent in our analysis, occurring in less than 2% of patients, and the incidence of skin toxicity was less common, whereas 13.6%, 29%, and 17–40% of patients experienced GI tract, hepatitis and skin toxicity, respectively, as previously reported (Wang et al., 2017; Suzman et al., 2018; Thompson et al., 2022). One underlying reason may be incomplete records in the electronic medical system and more patients receiving CTLA-4 inhibitor in clinical trials, as CTLA-4 inhibitors are associated with an increased risk of colitis and hepatitis compared to PD-1/PD-L1 inhibitors (Wang et al., 2017). This could lead to an underestimation of skin toxicity, colitis, and hepatitis in our analysis. In addition, patients included in this study usually received microecological agents, which may reduce the occurrence of colitis. Studies have shown that an increased baseline presence of Bacteroidetes species, such as *Bacteroidetes,* Bacteroidaceae*,* Rikenellaceae*, and* Barnesiellaceae, are linked to a reduced risk to colitis (Dubin et al., 2016; Aarnoutse et al., 2019; Som et al., 2019). Moreover, although the incidence of thyroid dysfunction was similar to those previously observed in a real-world setting, it was higher than that reported in a pooled analysis of many clinical trials (only with 5–10%) (Barroso-Sousa et al., 2018; Chang et al., 2019).

Importantly, we also found that there were certain differences between irAEs and ICI type. A resent meta-analysis looking at, the landscape and incidence of tr-AEs in a predominantly Chinese cohort, showed that the pooled incidence rates of all-grade TRAE (treatment-related adverse events) with sintilimab, camrelizumab, toripalimab, and pembrolizumab were 92.7%, 98%, 86.2%, and 74.1%, respectively. Moreover, the incidence of all-grade irAEs with sintilimab, camrelizumab, and toripalimab were 54.2%, 71.8% (RCCEP 58.6%), and 19.1%, respectively (Li et al., 2020). Additionally, a retrospective real-world study showed that the incidence rates of all-grade irAEs with sintilimab, camrelizumab, toripalimab, and pembrolizumab were 29.03%, 62.96% (RCCEP 22.22%), 9.52%, and 24.62%, respectively (Zheng et al., 2022). Furthermore, 147 articles and 23,761 cancer patients with 11 treated with different ICIs were included in a recent meta-analysis. Subgroup analysis showed that the frequency of all-grade and grade 3–5 irAEs with pembrolizumab were 67.25% and 16.58% (Song et al., 2020). Notably, our study found that sintilimab and camrelizumab had higher incidence of adverse reactions, and the irAEs of any grade and grade 1–2 irAEs were significantly different from toripalimab and pembrolizumab. However, standardized grade ≥3 irAEs of pembrolizumab was higher than those of sintilimab (13.21% vs. 7.12%, *p* = 0.026). Moreover, pembrolizumab had higher incidence of pneumonitis than other ICIs (sintilimab, toripalimab and camrelizumab), and the incidence was also higher than that previously reported in the literature (13.56% vs. 5%) (Nishino et al., 2016). This potential reason may be related to tumor type, for 55% of lung cancer and only <18% of melanoma in our study. As some studies have shown that many researchers support that there was higher incidence of pneumonitis but lower incidence of colitis in NSCLC and RCC compared with melanoma (Nishino et al., 2016; Wang et al., 2017). Further, pneumonitis was also the main reason for discontinuation in our study (38% of patients), where meta-analysis of pooled clinical trial data showed that 43% patients discontinued immunotherapy due to irAEs, with colitis being the most commonly reported reason for halting therapy (Kumar et al., 2017). this suggested that we should pay more attention or avoid using pembrolizumab for the people at high risk of developing pneumonia. In addition, in accordance with the literature, we found that the onset time of irAEs quite varied and our data highlight the correlation between incidence and onset time. We found that the median time of rare irAEs is later than that of other common irAEs (16.9 weeks vs. 5.9 weeks, *p* < 0.05), which suggests that we should be concerned about rare irAEs for patients on long-term treatment regimes.

Although early research shown that multiple factors were involved in the occurrence of irAEs, containing patient characteristics, medical history, medication history, ICIs type, therapeutic regimen and tumor type (Chennamadhavuni et al., 2022), there is still some debate about risk factors for irAEs. Some studies have reported that predictors for irAEs include concomitant chemotherapy, ICIs type, a higher body mass index, being female, C-reactive protein, neutrophil lymphocyte ratio, and smoking history (Suazo-Zepeda et al., 2021; Chennamadhavuni et al., 2022), whereas another study demonstrated no statistically significant irAE risk differences between the sexes (odds ratio [OR] = 1.19, 95% CI = 0.91–1.54, two-sided *p* = 0.21) (Jing et al., 2021). A real-world study also showed that serum albumin level ≥3.6 g/dl and history of Type I hypersensitivity reactions were risk factors for irAEs, but BIM, age and sex were not significant factors (Shimozaki et al., 2021). Although some studies showed that the incidence of irAEs was similar among different tumors, many researchers support that some irAEs was tumor-specific. As compared with melanoma, there was higher incidence of pneumonitis but lower incidence of colitis in NSCLC and RCC (Nishino et al., 2016; Wang et al., 2017). Additionally. Some studies indicated that pre-existing lung diseases increase the risk of irAE-pneumonitis (Shibaki et al., 2020), and patients with cardiovascular risk factors are more likely to occur cardiac-irAE (Pirozzi et al., 2021); whereas this is supported by conflicting evidence (Johns et al., 2020). In this study, we found that high eosinophil count, history of EGFR-TKI targeted therapy, cycles of ICI administration, and lung cancer were significantly correlated with the incidence of irAEs (*p* < 0.005), and cycles of ICI administration and lung cancer were independent risk factors for irAEs. In keeping with published clinical trial and meta-analysis data, these data suggest that we need to monitor the incidence of irAEs frequently for the people who have received EGFR-TKI therapy and are later receive ICIs treatment, especially the people with lung cancer. Interestingly, there were no significant difference between medical history, such as autoimmune condition, chronic pulmonary disease and history of thoracic radiation, and the development of irAEs; however, the relationship between preexisting autoimmune disorders and the development of irAEs remains controversial. With the increase in attention for irAEs, some studies showed that some baseline co-medications, such as antibiotics, PPI, diuretics and ACEI/ARD, could be associated with irAE. The underlying mechanisms was that altered anti-cancer activity to ICI were related to decreased incidence of irAEs, and gut microbial diversity was decreased by antibiotics (Kostine et al., 2021; Chennamadhavuni et al., 2022; Jing et al., 2022; Zhao et al., 2022). However, their clear relevance and the mechanism needs to be further studied in the future.

The correlation between irAEs and effective was not analyzed in our study due to confounding factors in the real world. However, based on the mechanism of action of ICIs, some published studies have shown that irAEs are associated with substantially improved ORR, DCR, PFS, and OS in patients who were treated with ICIs, and some experts consider irAEs to be a projection of the overall immune response to ICI therapy (Kobayashi et al., 2020; Kotwal et al., 2020; Shi et al., 2022). A prospective study showed that the development of vitiligo during melanoma treatment increases the likelihood of a positive clinical efficacy (Hua et al., 2016). Abu-Sbeih et al.,. performed a retrospectively studied the prognostic utility of the development of immune-mediated diarrhea and colitis in 173 patients with metastatic melanoma. The authors found that colitis was associated with improved OS and PFS, and higher grades of diarrhea were associated with even better patient’ OS rates. They also found that pancreatic adverse events were associated with worse PFS rates (Abu-Sbeih et al., 2019). A recent meta-analysis, which included 32 studies and 8,132 patients with NSCLC, melanoma, gastric cancer, renal cell carcinoma (RCC), urothelial carcinoma, and head and neck cancer (HNSCC), suggested that irAEs were, to some extent, a predictive factor for improved clinical outcomes and suggested irAEs as potential biomarkers for cancer patients undergoing ICI therapy (Park et al., 2021).

There are some limitations to the study, the main one being that it is a retrospective. All ICI-related ADRs were collected from the medical records, which could result in an over or underestimation of total irAEs. Further, there were some insufficient details in the description of clinical manifestations, and the detail duration of irAEs treatment was not available due to poor and incomplete medical records. In addition, the number of participants was relatively small and not symmetrical in different ICIs. Therefore, there were some bias may have been introduced based on the comparative analysis of spectrum and frequency of different ICI-induced toxicity, and the present findings should be interpreted with caution. Considering the complexity of participants, including, but not limited to, the medication, cancer type, and cumulative circles, etc. drug efficacy could not be evaluated objectively. Moreover, the analysis of risk factors could be not comprehensive. Thus, future studies are required to account for the bias and limitations included in this study.

## Conclusion

Based on our retrospective observational study, we made the following conclusions: (Vaddepally et al., 2020): RCCEP is a specific ADR of camrelizumab, and the incidence of irAEs is associated with ICI type. The overall incidence of irAEs following sintilimab and camrelizumab was higher and the incidence of pembrolizumab-induced pneumonitis was the highest. Therefore, it indicates that pneumonitis should be closely monitored during treatment. (Thompson et al., 2022). The onset of irAEs ranges widely, and the median onset time of rare irAEs is longer than that of other common irAEs (*p* < 0.05). This suggests that uncommon irAEs should be a concern for patients with undergoing long-term treatment. (Chen et al., 2021). Patients with lung cancer and more cumulative cycles have a higher probability of developing irAEs, and other risks of irAEs need to be further studied in the future.

## Data Availability

The datasets presented in this study can be found in online repositories. The names of the repository/repositories and accession number(s) can be found in the article/Supplementary Material.
