# Supplementary material for: PD-1 inhibitor-based adverse events in solid tumors: A retrospective real-world study
Source: Front Pharmacol. 2022 Nov 9;13:974376. doi: 10.3389/fphar.2022.974376 (PMC9681783; doi:10.3389/fphar.2022.974376)
Supplement: Supplementary file 1 [file Table1.DOCX]

**Table S1 Clinical characteristics of patients with different ICIs**

|  | **All patients**  **(n=362)** | **Sintilimab**  **(n=171)** | **Camrelizumab**  **(n=60)** | **Toripalimab**  **(n=72)** | **Pembrolizumab**  **(n=59)** | ***p*** |
| --- | --- | --- | --- | --- | --- | --- |
| **Sex** |  |  |  |  |  | 0.632 |
| Male | 275 (75.97%) | 126 (73.68%) | 49 (81.67%) | 54 (75.00%) | 46 (77.97%) |  |
| Female | 87 (24.03%) | 45 (26.32%) | 11 (18.33%) | 18 (25.00%) | 13 (22.03%) |  |
| **Age (year)** |  |  |  |  |  | 0.136 |
| < 65 | 193 (53.31%) | 87 (50.88%) | 35 (58.33%) | 33 (45.83%) | 38 (64.41%) |  |
| > =65 | 169 (46.69%) | 84 (49.12%) | 25 (41.67%) | 39 (54.17%) | 21 (35.59%) |  |
| **Smoke** |  |  |  |  |  | 0.125 |
| Yes | 222 (61.33) | 109 (63.74%) | 29 (48.33%) | 44 (61.11%) | 40 (67.80%) |  |
| on | 140 (38.67) | 62 (36.26%) | 31 (51.67%) | 28 (38.89%) | 19 (32.20%) |  |
| **chronic pulmonary disease** |  |  |  |  |  | 0.246 |
| Yes | 54 (14.92%) | 21 (12.28%) | 10 (16.67%) | 8 (11.11%) | 15 (25.42%) |  |
| on | 308 (85.08%) | 150 (87.72%) | 50 (83.33%) | 64 (88.89%) | 44 (74.58%) |  |
| **Treatment** |  |  |  |  |  | 0.624 |
| ICIs monotherapy | 60 (16.57%) | 28 (16.37%) | 9 (15.00%) | 10 (13.89%) | 13 (22.03%) |  |
| ICIs combinations | 302 (83.43) | 143 (83.63%) | 51(85.00%) | 62 (86.11%) | 46 (77.97%) |  |
| **Past medical history** |  |  |  |  |  |  |
| Targeted therapy history | 12 (3.31%) | 9 (5.26%) | 2 (3.33%) | 1 (1.39%) | 0 (0.00%) | 0.181 |
| Radiation history | 33 (9.12%) | 13 (7.60%) | 10 (16.67%) | 4 (5.56%) | 6 (10.17%) | 0.123 |
| Autoimmune condition | 3 (0.83%) | 3 (1.75%) | 0 | 0 | 0 | 0.646 |
| **Cumulative cycles** |  |  |  |  |  | 0.560 |
| ≤4 | 213 (58.84%) | 96 (56.14%) | 39 (65.00%) | 45 (62.50%) | 33 (55.93%) |  |
| >4 | 149 (41.16%) | 75 (43.86%) | 21 (35.00%) | 27 (37.50%) | 26 (44.07%) |  |
| **Type of cancer** |  |  |  |  |  | 0.001 |
| Lung | 201 (55.52%) | 118 (69.01%) | 20 (33.33%) | 29 (40.28%) | 34 (57.63%) |  |
| Other | 161 (44.48%) | 53 (30.99%) | 40 (66.67%) | 43 (59.72%) | 25 (42.37%) |  |

Table S2 Immune-related adverse events by organ category

|  | Any grade, n (%) | Grade 1-2, n (%) | Grade ≥3, n (%) |
| --- | --- | --- | --- |
| Thyroid dysfunction | 73 (20.17) | 73 (20.17) | 0 |
| Skin reaction | 36 (9.94) | 34 (9.39) | 2 (0.55) |
| Pneumonitis | 19 (5.25) | 2 (0.55) | 17 (4.70) |
| Infusion reaction | 9 (2.49) | 9 (2.49) | 0 |
| Myocarditis | 7 (1.93) | 4 (1.10) | 3 (0.83) |
| Reactive capillary hemangiomas (RCCEP) | 7 (1.93) | 7 (1.93) | 0 |
| Colitis | 6 (1.66) | 4 (1.10) | 2 (0.55) |
| Hepatitis | 6 (1.66) | 2 (0.55) | 4 (1.10) |
| Fever | 2 (0.55) | 2 (0.55) | 0 |
| Thrombocytopenia | 1 (0.28) | 0 | 1 (0.28) |
| Nephritis | 1 (0.28) | 1 (0.28) | 0 |
| Arthritis | 1 (0.28) | 1 (0.28) | 0 |
| Diabetes | 1 (0.28) | 0 | 1 (0.28) |
| Adrenal | 1 (0.28) | 0 | 1 (0.28) |
| Neurologic | 1 (0.28) | 1 (0.28) | 0 |
| Total | 171 (47.24) | 140 (38.67) | 31 (8.56) |

Table S3 Immune-related adverse events by ICIs regimen

|  | Whole colorts (n=362), n (%) | | | ICI combination (n=302), n (%) | | | ICI monotherapy (n=60), n (%) | | | P1 | P2 | P3 |
| --- | --- | --- | --- | --- | --- | --- | --- | --- | --- | --- | --- | --- |
|  | Any grade | Grade 1-2 | Grade ≥3 | Any grade | Grade 1-2 | Grade ≥3 | Any grade | Grade 1-2 | Grade ≥3 |  |  |  |
| Thyroid dysfunction | 73 (20.17) | 73 (20.17) | 0 | 68 (22.52) | 68 (22.52) | 0 | 5 (8.33) | 5 (8.33) | 0 | 0.012 | 0.012 | / |
| Skin reaction | 36 (9.94) | 34 (9.39) | 2 (0.55) | 33 (10.93) | 32 (10.60) | 1 (0.33) | 3 (5.00) | 2 (3.33) | 1 (1.67) | 0.161 | 0.078 | 0.208 |
| Pneumonitis | 19 (5.25) | 2 (0.55) | 17 (4.70) | 18 (5.96) | 2 (0.66) | 16 (5.30) | 1 (1.67) | 0 | 1 (1.67) | 0.296 | 1.000 | 0.411 |
| Infusion reaction | 9 (2.49) | 9 (2.49) | 0 | 6 (1.99) | 6 (1.99) | 0 | 3 (5.00) | 3 (5.00) | 0 | 0.360 | 0.360 | / |
| Myocarditis | 7 (1.93) | 4 (1.10) | 3 (0.83) | 5 (1.66) | 3 (0.99) | 2 (0.66) | 2 (3.33) | 1 (1.67) | 1 (1.67) | 0.727 | 0.517 | 0.420 |
| RCCEP | 7 (1.93) | 7 (1.93) | 0 | 1 (0.33) | 1 (0.33) | 0 | 6 (10.00) | 6 (10.00) | 0 | 0.000 | 0.000 | / |
| Colitis | 6 (1.66) | 4 (1.10) | 2 (0.55) | 6 (1.99) | 4 (1.32) | 2 (0.66) | 0 | 0 | 0 | 0.595 | 1.000 | 1.000 |
| hepatitis | 6 (1.66) | 2 (0.55) | 4 (1.10) | 5 (1.66) | 1 (0.33) | 4 (1.32) | 1 (1.67) | 1 (1.67) | 0 | 1.000 | 0.304 | 1.000 |
| Fever | 2 (0.55) | 2 (0.55) | 0 | 2 (0.66) | 2 (0.66) | 0 | 0 | 0 | 0 | 1.000 | 1.000 | / |
| Thrombocytopenia | 1 (0.28) | 0 | 1 (0.28) | 1 (0.33) | 0 | 1 (0.33) | 0 | 0 | 0 | 1.000 | / | 1.000 |
| Nephritis | 1 (0.28) | 1 (0.28) | 0 | 1 (0.33) | 1 (0.33) | 0 | 0 | 0 | 0 | 1.000 | 0.166 | / |
| Arthritis | 1 (0.28) | 1 (0.28) | 0 | 1 (0.33) | 1 (0.33) | 0 | 0 | 0 | 0 | 1.000 | 0.166 | / |
| Diabetes | 1 (0.28) | 0 | 1 (0.28) | 1 (0.33) | 0 | 1 (0.33) | 0 | 0 | 0 | 1.000 | / | 1.000 |
| Adrenal | 1 (0.28) | 0 | 1 (0.28) | 1 (0.33) | 0 | 1 (0.33) | 0 | 0 | 0 | 1.000 | / | 1.000 |
| Neurologic | 1 (0.28) | 1 (0.28) | 0 | 1 (0.33) | 1 (0.33) | 0 | 0 | 0 | 0 | 1.000 | 0.166 | / |
| Total | 171 (47.24) | 140 (38.67) | 31 (8.56) | 150 (49.67) | 122 (40.40) | 28 (9.27) | 21 (35.00) | 18 (30.00) | 3 (5.00) | 0.038 | 0.131 | 0.280 |

*P1: any grade irAEs between ICI combination and ICI monotherapy; P2: grade 1-2 irAEs between ICI combination and ICI monotherapy; P3: grade≥4 irAEs between ICI combination and ICI monotherapy*

Table S4A Immune-related adverse events between different ICIs (Any grade)

|  | Observed rate | | | |  | Standardized rate | | | |
| --- | --- | --- | --- | --- | --- | --- | --- | --- | --- |
|  | Sintilimab  (n=171) | Camrelizumab  (n=60) | Toripalimab  (n=72) | Pembrolizumab  (n=59) |  | Sintilimab  (n=362) | Camrelizumab  (n=362) | Toripalimab  (n=362) | Pembrolizumab  (n=362) |
| Thyroid dysfunction | 25.15 | 18.33 | 15.28 | 13.56 |  | 23.55a | 17.22a,b | 13.14b | 13.36b |
| Skin reaction | 12.28 | 8.33 | 8.33 | 6.78 |  | 12.83 | 7.22 | 7.09 | 6.82 |
| Pneumonitis | 5.26 | 3.33 | 2.78 | 10.17 |  | 4.60 | 5.55 | 2.95a | 9.80b |
| Infusion reaction | 4.09 | 1.67 | 0 | 1.69 |  | 3.29a | 1.11a,b | 0b | 1.63a,b |
| Myocarditis | 2.34 | 3.33 | 1.39 | 0 |  | 2.25a | 3.89a | 1.03a,b | 0b |
| RCCEP | 0 | 11.67 | 0 | 0 |  | 0 | 19.43 | 0 | 0 |
| Colitis | 2.34 | 0 | 2.78 | 0 |  | 2.62 | 0 | 2.07 | 0 |
| hepatitis | 2.34 | 0 | 1.39 | 1.69 |  | 2.25 | 0 | 1.91 | 1.78 |
| Fever | 0 | 1.67 | 0 | 1.69 |  | 0 | 1.11 | 0 | 1.63 |
| Thrombocytopenia | 0 | 0 | 1.39 | 0 |  | 0 | 0 | 1.03 | 0 |
| Nephritis | 0.58 | 0 | 0 | 0 |  | 0.47 | 0 |  | 0 |
| Arthritis | 0 | 0 | 0 | 1.69 |  | 0 | 0 |  | 1.63 |
| Diabetes | 0 | 0 | 0 | 1.69 |  | 0 | 0 |  | 1.63 |
| Adrenal | 0.58 | 0 | 0 | 0 |  | 0.47 | 0 |  | 0 |
| Neurologic | 0.58 | 0 | 0 | 0 |  | 0.47 | 0 |  | 0 |
| **Total** | **55.56** | **48.33** | **33.33** | **38.98** |  | **52.81** | **55.55** | **29.23** | **38.29** |

Table S4B Immune-related adverse events between different ICIs (Grade 1-2)

|  | Observed rate | | | |  | Standardized rate | | | |
| --- | --- | --- | --- | --- | --- | --- | --- | --- | --- |
|  | Sintilimab | Camrelizumab | Toripalimab | Pembrolizumab |  | Sintilimab | Camrelizumab | Toripalimab | Pembrolizumab |
| Thyroid dysfunction | 25.15 | 18.33 | 15.28 | 13.56 |  | 23.55a | 17.22 a,b | 13.14b | 13.36 b |
| Skin reaction | 12.28 | 5.00 | 8.33 | 6.78 |  | 12.83 | 5.00 | 7.09 | 6.82 |
| Pneumonitis | 1.17 | 0 | 0 | 0 |  | 0.94 | 0 | 0 | 0 |
| Infusion reaction | 4.09 | 1.67 | 0 | 1.69 |  | 3.29 | 1.11 | 0 | 1.63 |
| Myocarditis | 1.75 | 1.67 | 0 | 0 |  | 1.41 | 1.11 | 0 | 0 |
| RCCEP | 0 | 11.67 | 0 | 0 |  | 0 | 19.43 | 0 | 0 |
| Colitis | 1.75 | 0 | 1.39 | 0 |  | 1.78 | 0 | 1.03 | 0 |
| hepatitis | 1.17 | 0 | 0 | 0 |  | 0.94 | 0 | 0 | 0 |
| Fever | 0 | 1.67 | 0 | 1.69 |  | 0 | 1.11 | 0 | 1.63 |
| Thrombocytopenia | 0 | 0 | 0 | 0 |  | 0 | 0 | 0 | 0 |
| Nephritis | 0.58 | 0 | 0 | 0 |  | 0.47 | 0 | 0 | 0 |
| Arthritis | 0 | 0 | 0 | 1.69 |  | 0 | 0 | 0 | 1.63 |
| Diabetes | 0 | 0 | 0 | 0 |  | 0 | 0 | 0 | 0 |
| Adrenal | 0 | 0 | 0 | 0 |  | 0 | 0 | 0 | 0 |
| Neurologic | 0.58 | 0 | 0 | 0 |  | 0.47 | 0 | 0 | 0 |
| **Total** | **48.54** | **40.00** | **25.00** | **25.42** |  | **45.69** | **44.99** | **21.26** | **25.08** |

Table S4C Immune-related adverse events between different ICIs (Grade ≥3)

|  | Observed rate | | | |  | Standardized rate | | | |
| --- | --- | --- | --- | --- | --- | --- | --- | --- | --- |
|  | Sintilimab | Camrelizumab | Toripalimab | Pembrolizumab |  | Sintilimab | Camrelizumab | Toripalimab | Pembrolizumab |
| Skin reaction | 0 | 3.33) | 0 | 0 |  | 0 | 2.22 | 0 | 0 |
| Pneumonitis | 4.09 | 3.33 | 2.78 | 10.17 |  | 3.66 | 5.55 | 2.95 | 9.80 |
| Myocarditis | 0.58 | 1.67 | 1.39 | 0 |  | 0.84 | 2.78 | 1.03 | 0 |
| Colitis | 0.58 | 0 | 1.39 | 0 |  | 0.84 | 0 | 1.03 | 0 |
| hepatitis | 1.17 | 0 | 1.39 | 1.69 |  | 1.31 | 0 | 1.91 | 1.78 |
| Thrombocytopenia | 0 | 0 | 1.39 | 0 |  | 0 | 0 | 1.03 | 0 |
| Diabetes | 0 | 0 | 0 | 1.69 |  | 0 | 0 | 0 | 1.63 |
| Adrenal | 0.58 | 0 | 0 | 0 |  | 0.47 | 0 | 0 | 0 |
| **Total** | **7.02** | **8.33** | **8.33** | **13.56** |  | **7.12** | **10.55** | **7.97** | **13.21** |

Table S5 Clinical characteristics and outcomes between different irAEs

| irAEs | Signs and symptoms | Management | Outcome | Continue immunotherapy |
| --- | --- | --- | --- | --- |
| **Thyroid dysfunction** | | | | |
| Grade 1-2 (73) | Most patients have no symptom, possible lethargy, sensation of being cold and fatigue;  Some patients transition from hyperthyroidism to hypothyroidism | close monitoring (n=40); Propranolol (n=1); Levothyroxine (n=31); Prednisone + Levothyroxine (n=1) | improved | Yes |
| **Skin reaction** | | | |  |
| Grade 1-2 (34) | Rash, with or without pruritus, some patients have skin lesions or blisters, vitiligo observed by one patient with melanoma.  Grade 1-2 covering ≤30%; body surface area (BSA) and Grade ≥3 covering ≤30% BSA with or without symptoms | H2RA +topical steroids (n=34) | improved | yes |
| Grade ≥3 (2) |  | Prednisolone + H2RA +topical steroids (n=2) | improved | No (n=2) |
| **Pneumonitis** | | | |  |
| Grade 1-2 (2) | No symptoms, only with lung imaging changes | close monitoring (n=2) | improved | No |
| Grade ≥3 (17) | New cough, fatigue, shortness of breath or hypoxia; with lower oxygen saturation and lung imaging changes | Methyl-prednisolone (n=17) | improved (n=12); rebound (n=4);  died (n=1) | No |
| **Infusion reaction** | | | |  |
| Grade 1-2 (9) | Faster heart rate, mouth dryness, shortness of breath, dizziness, fever/chills | Slowing down infusion speed (n=1); NSAIDs (n=6); close monitoring (n=2) | improved | Yes (n=7); No (n=2) |
| **Myocarditis** | | | |  |
| Grade 1-2 (4) | No symptoms, only with abnormal cardiac biomarkers | Holding immunotherapy, trimetazidine / creatine phosphate) (n=2) | improved | Yes (n=2); No (n=2) |
| Grade ≥3 (3) | Shortness of breath, arrhythmia, chest pain, with abnormal cardiac biomarkers (troponin I and T, creatine kinase) and inflammatory biomarkers (ESR, CRP) | Methylprednisolone + creatine phosphate/adenosine cyclphosphate (n=3) | improve (n=1);  died (n=2) | No |
| **Reactive capillary hemangiomas** | | | |  |
| Grade 1-2 (7) | red hemangioma occurred on the face, neck, trunk, and four limbs for many patients, one patient occurred on lips | Hemostatic (Yunnan Baiyao, n=2); H2RA (n=2); close monitoring (n=3) | improved (n=4); unchanged (n=3) | No |
| **Colitis** | | | | |
| Grade 1-2 (4) | Diarrhea, With or without abdominal pain and hematochezia | Montmorillonite Powder (n=3); Montmorillonite Powder + Prednisone (n=1) | improved (n=3); rebound (n=1) | No |
| Grade ≥3 (2) |  | Montmorillonite Powder + Prednisone (n=2) | improved | No |
| **Hepatitis** | | | | |
| Grade 1-2 (2) | No symptoms, with elevation of transaminitis (ALT and AST), possible elevation of bilirubin | Hepatoprotective drugs (n=2) | improved | Yes (n=2) |
| Grade ≥3 (4) |  | Hepatoprotective drugs + methylprednisolone (n=4) | improved | No (n=4) |
| **Fever** | | | | |
| Grade 1-2 (2) | Fever without chills (T 39.3℃)，with normal inflammatory biomarkers (WBC, Neu#, CRP and PCT) | Ibuprofen + Prednisone | improved | No |
| **Thrombocytopenia** | | | | |
| Grade ≥3 (1) | No symptoms, with thrombocytopenia | Recombinant human thrombopoietin + infusing platelet | died | No |
| **Nephritis** | | | | |
| Grade 1-2 (1) | Elevation of creatinine and blood urea nitrogen | delayed treatment | improved | Yes |
| **Arthritis** | | | | |
| Grade 1-2 (1) | joint pain on right knee | NSIADs+ Prednisone | improved | Yes |
| **Diabetes** | | | | |
| Grade ≥3 (1) | Thirst, frequent urination, with high blood sugar | Oral hypoglycemic drugs + injecting insulin | improved | No |
| **Adrenal** | | | | |
| Grade ≥3 (1) | Cognitive impairment, vomiting, lethargy, fatigue, sensation of being cold; with abnormal cortisol function | methylprednisolone | improved | No |
| **Neurologic** | | | | |
| Grade 1-2 (1) | Numbness on both feet, possible stabbing pain; with imaging changes of the central nervous system | Vit B1、B12 | improved | Yes |

a: chest CT: pulmonary lesions, with uneven density, diffuse fine shadows, or streak shadows, or patchy shadows, or bilateral ground glass density shadow; H2RA: H2-receptor blockers; NSAIDs: ESR: CRP: WBC, Neu#; PCT; ALT and AST
